# The Mark of the Cognitive and the Coupling-Constitution Fallacy: A Defense of the Extended Mind Hypothesis

**DOI:** 10.3389/fpsyg.2017.02061

**Published:** 2017-11-28

**Authors:** Giulia Piredda

**Affiliations:** Istituto Universitario di Studi Superiori di Pavia (IUSS), Pavia, Italy

**Keywords:** extended mind, mark of the cognitive, coupling-constitution fallacy, functionalism, methodological individualism

## Abstract

Clark and Chalmers (1998) introduced the extended mind hypothesis, according to which some mental states can be realized by non-biological external resources. A lively debate has flourished around this hypothesis, connected with the issues of embodiment, embeddedness, situatedness and enaction (cf. Clark, 2008; Menary, 2010; Shapiro, 2011). Two of the main criticisms addressed to the functionalist version of the extended mind thesis have been the so-called “coupling-constitution fallacy” and the alleged lack of a mark of the cognitive (Adams and Aizawa, 2001, 2005, 2009, 2010a,b). According to Adams and Aizawa, extended cognition is a logical possibility, but is not instantiated in our world. Following this view, they defend a “contingent intracranialism,” based on a specific mark of the cognitive that they propose. In this paper I intend to show that neither criticism is effective against the extended cognition thesis. In particular:

- the mark of the cognitive proposed by Adams and Aizawa does not secure contingent intracranialism;
- the coupling-constitution fallacy criticizes extended cognition on precisely the point the theory was intended to defend: namely, that the best way to individuate cognitive systems, given a minimal mark of the cognitive, is to rely on coupling relations between agents and environmental resources.

## Introduction

“Where does the mind stop and the rest of the world begin?” This is the starting point of the reflection by Clark and Chalmers (1998) on the extended mind. According to their view, the boundaries of our mind can vary depending on the activities we are engaged in. What they mean by this is not only that we are embodied agents—a fact that reflects on our cognitive activities –, but also that some of our dispositional mental states, such as beliefs, or even desires, can be realized in external vehicles such as a notebook or computer file. When we use the external environment as an extension of our space for thinking, some of our cognitive processes can also be considered extended: when we use pen and paper, for instance, to perform a multiplication, following well-known heuristics learned at school (Clark, 1997, 2003, 2007, 2008, 2010a,b, 2011; Wilson, 2004; Wilson and Clark, 2009). Obviously, this is not always the case. Sometimes our minds are bound to our brain structure: when we dream, for example, or when we think without interacting with the external environment.

The debate about extended mind and cognition has flourished in recent decades, connecting the issue to embodiment, embeddedness, situatedness and enaction (Noë, 2004; Clark, 2008; Robbins and Aydede, 2009; Menary, 2010; Shapiro, 2011).

In this paper I will focus exclusively on the functionalist version of the extended mind originally proposed by Clark and Chalmers (1998), and further developed mainly by Clark (2003, 2007, 2008, 2010a,b, 2011) and Wilson (2004).

Two of the main criticisms addressed to the functionalist version of the extended mind have been the so-called “coupling-constitution fallacy” and the alleged lack of a mark of the cognitive (Adams and Aizawa, 2001, 2005, 2009, 2010a,b). According to Adams and Aizawa, extended cognition is a logical possibility, but is not instantiated in our world. Following this view, they defend a “contingent intracranialism,” based on a specific mark of the cognitive they propose.

In this paper I intend to show that neither criticism is effective against extended cognition. In particular:

- the mark of the cognitive proposed by Adams and Aizawa does not secure contingent intracranialism;
- the coupling-constitution fallacy criticizes extended cognition on precisely the point the theory was intended to defend: that, given a minimal mark of the cognitive, coupling relations should be the main guide to individuating cognitive systems.

In the next section I will introduce the main theses pursued by supporters of the extended mind. In section The Coupling-Constitution Fallacy and the Mark of the Cognitive I will present the two criticisms put forward by Adams and Aizawa. This section will also be devoted to my criticisms of the mark of the cognitive proposed by Adams and Aizawa, which in my view fails to justify their position (“contingent intracranialism”) and is not effective against extended cognition. In section Criticism of the Coupling-Constitution Fallacy I will analyze the so-called “coupling-constitution fallacy,” concluding that in response to this criticism, extended cognition supporters should “bite the bullet” and claim that “coupling-constitution” is not a fallacy at all: it is their positive thesis about how cognitive systems should be individuated. As I argue in section Coupling, Constitution, and Taxonomical Practices in Cognitive Science, it derives from the rejection of individualism as a methodological strategy to individuate cognitive systems. Once the actual possibility of extended cognition has been accepted, and the traditional boundaries of the individual renounced, the observation of coupling relations is the fundamental guide in the individuation of cognitive systems—extended or otherwise. Section Conclusion: Metaphysics, Epistemology and the Mark of the Cognitive will close the paper with some final reflections on the relation between metaphysics and epistemology in the extended mind framework.

### The extended mind: a problematic boundary

From an argumentative point of view, the extended mind thesis was originally defended by means of a famous thought experiment in which two characters have the intention to go to a museum (Clark and Chalmers, 1998; Menary, 2010, p. 12 ff). The first character, Inga, has an intact cognitive apparatus: she retrieves the address from her memory, and reaches the museum. The other, Otto, suffers from a mild form of Alzheimer's disease that impairs his mnemonic capacity: for this reason he carries a notebook in which he constantly writes down any new information he is certain about. It is like an external memory that he must refer to prior to undertaking any activity. Thus, Otto consults his notebook, retrieves the address and reaches the museum. Now, the question is: is there a substantial difference between the structure of Otto's actions and those of Inga? Clark and Chalmers claim that there is not. In particular, they maintain that it is possible to ascribe to both characters, before they engage in the act of retrieval, a dispositional belief about the museum address. The two dispositional beliefs, Otto's and Inga's, would have the same content (i.e., the museum address) but different kinds of vehicles (i.e., biological memory for Inga, a piece of paper for Otto).

From this brief presentation, it seems clear that the philosophical basis of the idea of extended mind comprises a form of externalism—namely *active or vehicular externalism*—and a functionalist view of the mind. According to vehicular externalism, environmental (external) resources not only play an active role in triggering and driving cognitive and mental processes, but under certain conditions should be considered as part of those same processes, plausibly qualifying as proper thought vehicles. This position is defended in virtue of the parity principle, which clearly expresses Clark and Chalmers' anti-internalistic position:

If, as we confront some task, a part of the world functions as a process which, were it done in the head, we would have no hesitation in recognizing as part of the cognitive process, then that part of the world is (so we claim) part of the cognitive process (1998, p. 8).

The individuation of mental states as functional states is also an important component of the “Otto and Inga” thought experiment. According to functionalism, mental states are to be individuated through an analysis of the typical causal relations they instantiate. For example, pain has been defined as the state provoked by some sort of injury that normally causes complaints and the desire to eliminate it. In this sense, *extended* functionalism is the doctrine according to which “externally located objects, such as pen and paper, can be exploited in problem solving to form proper parts of a cognitive process” because they play the right kind of functional role in a cognitive or mental routine (Kiverstein and Clark, 2009, p. X; Wheeler, 2010). That is, extended functionalism individuates cognitive processes and mental states through the observation of causal chains between individual and environmental resources (e.g., external memory). In this view, “[c]ognitive processing can sometimes include operations and capacities provided by the extraorganismic environment” (ibidem)^1^.

In this revisionary metaphysics, it is important to find a way to distinguish proper cognitive extensions from mere contingency relations between a biological organism and an external resource. To this end Clark and Chalmers suggest that we generalize some central features of the Otto case:

1. Otto never acts without consulting the notebook (constant presence and automatic use),
2. The information in the notebook is directly and easily available,
3. Otto automatically endorses information retrieved from the notebook,
4. The information in the notebook has been consciously endorsed in the past^2^.

These criteria should guide our individuation of genuine cases of extended cognition, also described through the concept of “coupling.” In the dynamical systems theory, two systems are *coupled* when the variables of the first vary following the variables of the other (Kelso, 1995; van Gelder, 1995, 1998). Cases of coupled systems in the domain of extended cognition are described in Clark's words:

These are cases when we confront a recognizably cognitive process, running in some agent, that creates outputs (speech, gesture, expressive movements, written words) that, recycled as inputs, drive the cognitive process along. In such cases, any intuitive ban on counting inputs as parts of mechanisms seems wrong (Clark, 2008, p. 131).

This kind of coupling creates a virtuous circle by which the entire system improves its performance by producing outputs that are then recycled as inputs for the system, generating what has been called a process of “information self-structuring” (ibidem; Shapiro, 2011, pp. 175–176). The problem now, in the case of Otto and his notebook, is: what is the difference between coupled components as causal contributors to a process and its real constituents? In virtue of what principle does something enter the ontology of a process or system? The coupling-constitution fallacy, a criticism addressed to the extended mind thesis by Adams and Aizawa (2001, 2005, 2009, 2010a,b) and to which we now turn our attention, deals with these questions.

## The coupling-constitution fallacy and the mark of the cognitive

According to the extended mind hypothesis, an external resource can be considered as part of the cognitive or mental apparatus of an individual, as long as the relation between the two displays the features described above. Adams and Aizawa think that this position reflects an essential philosophical weakness, which they discuss in the so-called “coupling-constitution fallacy” (Adams and Aizawa, 2001, 2010a,b; Aizawa, 2010). The criticism goes as follows: the fact that it is always available, reliable and constantly used does not make a given resource cognitive or mental *per se*. In particular, what does not follow is the cognitive (or mental) nature of that particular resource. They write:

The fact that object or process X is coupled to object or process Y does not entail that X is part of Y. e.g., The neurons leading into a neuromuscular junction are coupled to the muscles they innervate, but the neurons are not a part of the muscles they innervate (Adams and Aizawa, 2010a, p. 68).

It seems reasonable: neurons and muscles are coupled, but this does not necessarily imply that one is a part of the other. Moreover, it is not because they are coupled that we can call muscles an extension of the neural system, or, vice versa, neurons an extension of the muscular system. However, it is true that neurons and muscles can be considered parts of the same *system*, an “extended neuromuscular system,” or taking part in the same *process*, an “extended neuromuscular process^3^”.

Adams and Aizawa go further: “If the fact that an object or process X is coupled to a cognitive agent does not entail that X is a part of the cognitive agent's apparatus, what does? The nature of X, of course. One needs a theory of what makes a process a cognitive process, rather than a non-cognitive process. One needs a theory of the mark of the cognitive” (Adams, 2010; Adams and Aizawa, 2010a, p. 68).

According to Adams and Aizawa, then, the extended view of the mind lacks a proper “mark of the cognitive”, some specific condition that individuates the presence of a unified cognitive process or system. It is widely accepted that cognitive processes must include some form of information processing, but the question of the distinctive feature of the cognitive is still on the table. This problem concerns the principle of individuation of cognitive scientific entities, according to accepted scientific practice.

But what is the position of Adams and Aizawa on this subject? Having briefly summarized their two main criticisms of the extended mind, I will now present their position on the mark of the cognitive. The next section will be devoted to a discussion of their proposal.

In order to define what a cognitive process is—or, better, what makes a process a *cognitive* process—Adams and Aizawa propose two hypotheses^4^, one conceptual and one empirical. These hypotheses make them “defenders of orthodoxy,” concluding that “there are principled reasons for believing that the kind of cognitive processing cognitive psychologists care about is, essentially without real-world exceptions, intracranial” (Adams and Aizawa, 2010a, p. 9). The following define what Adams and Aizawa call their “mark of the cognitive”:

- cognitive processes involve non-derived representations, that is representations that mean what they mean independently of other representational or intentional capacities;
- cognitive processes are those that take place in virtue of certain mechanisms (Adams and Aizawa, 2010a, p. 9).

It is important to notice that Adams and Aizawa intend their intracranialism to be *contingent*, because they want to accept, as a mere metaphysical or conceptual possibility, that cognition could be extended, even though they argue that such is not the case in our world. Denying the possibility of extended cognition *a priori*, in fact, would represent a problem for the functionalist framework that they seem to accept. In other words, they want to maintain a Modal thesis of Extended Cognition (MEC), a consequence of many functionalist views of cognition: “It is possible that cognition extends into the body and surrounding environment,” and attempt to reject a thesis of Actually Extended Cognition (AEC): “Cognition extends into the body and surrounding environment” (Adams and Aizawa, 2010a, p. 25).

### Criticisms of Adams and Aizawa's mark of the cognitive

In this section I will present some reasons for dissatisfaction with the mark of the cognitive proposed by Adams and Aizawa, which, I will attempt to show, is also ineffective against the extended cognition thesis. A general point concerns the force of the criticism, which is to some degree weakened by an observation made by Adams and Aizawa themselves. Indeed, they write:

in all fairness to Clark and other extended mind theorists, it must be admitted that one of the shortcomings of contemporary cognitive psychology is that there is no well-established theory of just exactly what constitutes the cognitive (2010b, p. 68).

Thus, in admitting that the problem affecting extended mind theories affects contemporary cognitive psychology in general^5^, they grant a somewhat mitigating circumstance to their opponents. Nevertheless, they persist in their argument against extended cognition.

In the following, I will reconstruct the line of argumentation that, in the vision of the authors, leads to the conclusion of a “contingent intracranialism”:

(C) “cognitive psychologists have one principled reason to think that cognition is typically intracranial” (Adams and Aizawa, 2010a, p. 9).

I will offer reasons to doubt (A) and (B) as valid criteria to individuate cognitive processes.

#### Against (A)

Starting from (A), we can reconstruct a briefly sketched argumentation that should lead to “contingent intracranialism”:

(A) “cognitive processes involve non-derived representations, that is representations that mean what they do independently of other representational or intentional capacities,”
(A.1) “because these representations are typically found inside, but not outside, the brain,”
(C) “cognitive psychologists have one principled reason to think that cognition is typically intracranial” (Adams and Aizawa, 2010a, p. 9).

The first observation regards the notion of non-derived representation, traditionally used in the debate about intentionality. According to some philosophers (e.g., Searle, 1980, 1992), there is a difference between “derived intentionality,” displayed by signs that owe their meaning to an act of interpretation, such as traffic lights, flags and words, and “intrinsic (or non-derived) intentionality,” characteristic of a naturalistic and non-conventional way of meaning. Mental states are considered a typical example of this latter kind of intentionality, which also constitutes a possible mark of the mental (cf. the usual reference is Franz Brentano).

Some philosophers, however, do not accept the distinction between intrinsic and derived intentionality, arguing that it is not among the clearest in the recent history of philosophy (Clark, 2005, p. 4). Even if it could, in principle, be considered clear, there still could be serious difficulties in discriminating sophisticated but significant cases, “such as robots, whose trajectories can unfold without any direct dependence on us, their creators, and whose discriminations give their internal states a sort of meaning to them that may be unknown to us and not in our service” (Dennett, 2009; also Dennett, 1971, 1990; Shapiro, 2009, p. 271, on the origin of original intentionality).

The real problem of condition (A), though, is not only the potential lack of clarity of the notion of original intentionality. The fact is that the appeal to non-derived representations does not rule out extended cognition. Extended mind theorists, in fact, have never claimed “that one could build an entire cognizer out of Otto-style notebooks” (Clark, 2005, p. 6). They have never denied the role of the brain in the unfolding of cognitive processes; thus, they have no problems admitting that, for every cognitive process, there will be some non-derived representations at work.

Given that, there would be a problem if Adams and Aizawa maintained that every representation involved in a cognitive process should be of the non-derived kind. Thus, Clark wonders:

The question is, must everything that is to count as part of an individual's mental processing be composed solely and exclusively of states of affairs of this latter intrinsically content-bearing kind? I see no reason to think that they must (Clark, 2010a, p. 48).

Indeed, this is not the case, as Adams and Aizawa write: “it is unclear to what extent each cognitive state of each cognitive process must involve non-derived content” (Adams and Aizawa, 2001, p. 50; cit. in Adams and Aizawa, 2010b, p. 69).

This second concession by Adams and Aizawa renders their requirement of non-derived representations in cognitive process ineffective against extended cognition. Within the framework of extended cognition it is perfectly acceptable to claim that every extended cognitive process involves non-derived representations.

In the end, the requirement that cognitive processes involve non-derived representations does not rule out extended cognitive processes.

#### Against (B)

Other problems concern (B), according to which “cognitive processes are those that take place in virtue of certain mechanisms”. As previously stated, Adams and Aizawa maintain the Modal thesis of Extended Cognition (MEC): “It is possible that cognition extends into the body and surrounding environment”; thus, they grant that “these mechanisms could occur outside of the brain”. Nevertheless, since they typically do not, we are led to the usual conclusion: (C) “cognitive psychologists have one principled reason to think that cognition is typically intracranial” (2010a, p. 9).

The mechanisms Adams and Aizawa refer to in (B) are those investigated by cognitive psychology, such as those underlying short-term memory or understanding. As we know, these mechanisms realized by the human brain have some typical characteristics. For example, human short-term memory has a specific “size capacity.” Human subjects are generally capable of remembering strings of five, six or seven letters, and this is known in cognitive psychology as Miller's rule. Now, even if they do not intend to propose that “in order to be short-term memory, something must respect Miller's rule”—which would mean renouncing the functionalism entailed in MEC –, Adams and Aizawa nevertheless maintain that “findings such as this should guide us in determining what memory is like and what really differentiates cognitive processes and mechanisms from non-cognitive processes and mechanisms” (2010a, p. 10).

There are at least two problems with this position. First, in order to preserve MEC, the specific characteristics of the mechanisms underlying human cognitive processes should not, in my opinion, be taken as a strict guide in distinguishing cognitive from non-cognitive processes. These are, in fact, characteristics of the typical human/biological expression of those cognitive processes. If, for example, an animal instantiates versions of memory that do not share the same characteristics as human memory, should it be excluded from the domain of the cognitive? I believe that, in order to preserve MEC, the cognitive processes to be explained should be described in a sufficiently broad manner as to possibly include several kinds of expressions of those same processes. Viewed from this perspective, memory may be seen as the capacity to retain certain elements, independently of the quantitative capacity of the individual. As Levin (2008) says: “what makes something a mental state of a particular type does not depend on its internal constitution, but rather on the way it functions, or the role it plays, in the system of which it is a part.” This is a problem not only for Adams and Aizawa, but also for Robert Rupert, who criticizes extended cognition (also) on the basis of the differences—significant, according to him—between, e.g., biological and artificial memory (2004). Will we be forced to accept that without the typical idiosyncrasies of our kind of memory we cannot speak about memory at all? What about animal cognition? Artificial cognition^6^?

Secondly, in seeking to define what qualifies as cognitive I suspect that reference to what is considered “typical” is not a good strategy, unless one intends to maintain an “orthodox” attitude that precludes further discussion. Of course, cognitive psychology is a healthy discipline, but this does not mean that we must conflate the “typical” with the “normative,” as I think Adams and Aizawa do. To look at the typical in a paradigmatic way can, in general, be a good guide to arriving at an idea of which phenomena a certain discipline deals with. We could do the same with physics, biology or history, if we were unfamiliar with these disciplines. But this could hardly be considered a normative criterion to individuate the precise object of study of a discipline we know well. If today's cognitive psychology focuses only on the processes performed by natural cognitive agents, does this mean that we should stop wondering whether artificial or animal cognition can exist? I doubt this would be a reasonable strategy, particularly in a functionalist (or MEC preserving) framework.

Summing up, we have seen that the conditions Adams and Aizawa choose to mark the cognitive:

- appeal to the distinction between intrinsic and derived intentionality, which is questionable,
- do not—as they refer to what is typical for cognition—rule out extended cognition, and confuse the typical with the normative,
- pose some problems for the Modal thesis of Extended Cognition (MEC), which Adams and Aizawa are willing to maintain.

Now, even if the mark of the cognitive proposed by Adams and Aizawa is less than convincing, the coupling-constitution fallacy might still be considered an effective criticism against the extended mind thesis. If this were the case, Adams and Aizawa would merely have to identify other conditions to mark the cognitive. In the remainder of the paper, I will attempt to show that even the coupling-constitution fallacy is not a powerful criticism against the extended mind.

As we have seen, within the extended cognition framework coupling plays a fundamental role in individuating the extended systems to which cognitive properties can be ascribed, e.g., Otto and his notebook. If the kind of coupling satisfies the criteria proposed by Clark and Chalmers, what other features do we need in order to define a certain system as “cognitive”? I believe that, unless one endorses some sort of methodological individualism according to which the boundaries of the cognitive system are already settled from the start, then we have to admit that attributing to coupling a fundamental role in individuating cognitive systems is exactly what the supporters of extended cognition mean to defend. Thus, criticizing them on this basis misses the point. In the remainder of the paper, I will articulate this criticism, attempt to defend extended cognition from it, and will make some further observations on coupling and the mark of the cognitive.

## Criticism of the coupling-constitution fallacy

In this section I will present an analysis of the criticism known as coupling-constitution fallacy, connected to the discussion about the mark of the cognitive^7^. Let us first review the point by Adams and Aizawa.

According to Adams and Aizawa, the fallacious argument consists in deriving that “X is part of Y” from the fact that “object or process X is coupled to object or process Y” (Adams and Aizawa, 2010a, p. 68)^8^. Another way to put it, slightly different but substantially analogous, is the following:

(CCF2) “The pattern of reasoning here involves moving from the observation that process X is in some way causally connected (coupled) to a process Y of type Z to the conclusion that X is part of a process of type Z” (Adams and Aizawa, 2009, p. 83).

In the case of Otto, the correction of the fallacious argument would be: The fact that Otto's notebook is coupled to the cognitive agent Otto does not entail that the notebook is a part of the cognitive agent Otto. In other words, coupling relations are not sufficient for cognitive extension to obtain. But how is the relation between coupling and constitution conceived in the extended mind framework? Of course, the validity of the allegedly fallacious argument depends on the relation one conceives between coupling and constitution, or between coupling and extension. Thus, it is on this relation that I will focus my attention.

I will argue that, given the relation between coupling and constitution in the extended mind framework, the so-called coupling-constitution fallacy is not a fallacy at all; rather, it is basically a restatement of the positive thesis claimed by extended cognition, which has its roots in the anti-individualistic attitude adopted in cognitive science taxonomy.

It must be admitted that, taken at face value, the fallacy argument seems to correctly point out that a coupling relation is not the same as a constitutive relation, a position Adams and Aizawa attribute to the supporters of extended cognition. Intuitively, they might be right on this: coupling and constitution are conceptually distinct, and the thesis that one kind of relation entails the other is to be claimed only at one's own risk. In this case, however, it is a viable option, as Kagan and Lassiter (2013) point out. The claim that “coupling relations are distinct from constitution relations” does not imply that “the former do not entail the latter”:

[I]f property P1 is distinct from property P2, it does not follow that ‘being P1’ fails to nomologically or logically entail ‘being P2’. For example, ‘being a renate’ is distinct from ‘being a chordate’ but that itself does not bar ‘being a renate’ from nomologically entailing ‘being a chordate’ (2013, p. 183).

In other words, even if it is (trivially) true that coupling relations are distinct from constitution relations, this does not exclude *per se* that the first kind of relation might be, for example, a reliable clue to finding the other kind, or even a mark, given some previous condition, of the presence of the other kind. As a matter of fact, I believe that something similar is at play in the framework of extended cognition, as we will see below.

### Coupling, constitution, and taxonomical practices in cognitive science

To introduce my interpretation of the coupling-constitution fallacy, I would point out that what is at stake here is the way in which cognitive psychology, or cognitive science, should construe its taxonomies and define the boundaries of its objects of study: what are the crucial elements on the basis of which cognitive psychology individuates cognitive systems?

For reasons connected to the history of cognitive science, the most frequent position concerning the way in which psychology should construe its taxonomies has been methodological individualism, which has been supported and defended in various forms (cf. Fodor, 1980; Stich, 1983). The basic idea was that in order to study cognition one could abstract away from the fact that cognitive agents live in a physical and social environment. Every significant difference in an environment would be reported in some variation of the intrinsic physical states of the individual, which constituted the sufficient basis to be taken into account in psychological analysis. Thus, individualism led to a restrictive methodology (Wilson, 1995, 2004).

Thus, classical cognitive science has traditionally assumed an individualistic stance, for reasons ranging from the influence of Cartesianism to the need for local, syntactic computations^9^. According to individualism, the psychological properties of an individual supervene on her intrinsic physical properties, the repository of her causal powers. Therefore, in an individualistic framework:

there is no psychological difference without a corresponding difference in the intrinsic, physical states of the individual (Wilson, 2004, p. 94).

Individualism, as well as its opponent, taxonomic externalism^10^, are theses about the metaphysical determination of psychological states, i.e., about which elements are relevant in order to define the psychological state of a given individual. On this issue, they hold opposite positions:

Individualists claim, and externalists deny, that what occurs inside the boundary of an individual metaphysically determines the nature of that individual's mental states (Wilson, 2004, p. 80).

But that what occurs *outside* the boundary of an individual is metaphysically relevant to determining the nature of that individual's mental states is exactly what the kind of externalism supported by Clark and Chalmers, that is vehicular externalism, supposes. The idea that causal coupling, i.e., causal interaction with the environment, not only has an effect on our mental content and psychological life, but also helps determine the metaphysical boundaries of the individuals studied by cognitive psychology, is precisely the idea upon which the example of Otto is based. It seems, then, that vehicular externalism implies taxonomic externalism, and with it the negation of individualism in the construction of psychological taxonomies^11^. Thus, the fact that in the extended cognition framework the observation of causal coupling guides the metaphysical determination of an individual's boundaries, that is its constitution, is exactly what the supporters of extended cognition mean to defend.

As a matter of fact, the whole “extended cognition movement” has a strong anti-individualistic tension at its roots, based on various reasons for dissatisfaction with individualism that have emerged over the years. Are internal resources really sufficient to explain our cognitive performance? Is the brain all there is to cognition? Part of the insight of the extended cognition thesis has been to “shake” the individualistic attitude by introducing the notion that some resources external to the individual can play an important role in an agent's cognitive life: a role not necessarily reflected in her internal states (see the case of Otto's notebook as a repository of his dispositional beliefs). Following this intuition, the supervenience base of the cognitive life of an agent is extended to all possible elements that are coupled to her cognitive processes. Without these elements, her cognitive life would not be the same. Thus, this anti-individualistic position breaks the “invisible rule” of considering the internal as the exclusive location of the cognitive processes, even if it remains the privileged one (cf. Clark, 2008, p. 116 ff.)^12^. Within the extended cognition framework, even external resources can enter the magical realm of the cognitive. And how do we individuate the external resources relevant to the determination of cognition? Once biological boundaries are no longer seen as playing the only specific role in the individuation of the boundaries of cognitive systems, the observation of the coupling relations between agent and environment becomes central to this taxonomical process.

When Clark and Chalmers specify the criteria through which we can recognize “extended mental or cognitive states,” they are referring to the idea of particularly tight causal relations, or coupling. It is through the observation of the causal links between different entities—what they call the “causal dynamics”—that we are guided in the individuation of extended vehicles of mental content—and thus extend cognition. About the role of coupling Clark writes:

Let us first be clear then about the precise role of the appeal to coupling in the arguments for the extended mind. The appeal to coupling […] is intended to make some object, which in and of itself is not usefully (perhaps not even intelligibly) thought of as *either cognitive or noncognitive*, into a *proper part of some cognitive system*, such as a human agent. It is intended, that is to say, to ensure that the putative part is poised to play the kind of role that *itself* ensures its status as part of the agent's cognitive routines (Clark, 2010b, p. 83).

In conclusion, giving up the individualistic constraint is a crucial step in the path toward the revisionary metaphysics proposed by extended cognition, one that makes the passage from coupling to constitution far more reasonable than it might initially appear. It is for this reason that I do not find the coupling-constitution fallacy an effective criticism against extended cognition: given the anti-individualism inherent in the extended cognition thesis, an endorsement of the coupling-constitution implication is a perfectly viable and reasonable position.

Therefore, within the framework of extended mind, causal coupling is a fundamental guide for individuating constitutive relations and, thus, cognitive systems. What drives extended mind supporters in the search for this innovative ontology is a need for the construction of better explanations: explanations that are able to convey the complex connections between biological, social and cultural elements in defining our minds. Constitutive relations are thus individuated on the ground of epistemological considerations concerning the search for the best explanation. Therefore, coupling, together with some epistemological concerns regarding the best way to explain a certain phenomenon, will be responsible for the individuation of an innovative ontology. As a matter of fact, extended mind supporters propose a revision of our mental ontology in line with epistemological considerations about our best explanations:

We do not intend to debate what is standard usage; our broader point is that the notion of belief *ought* to be used so that Otto qualifies as having the belief in question. […] By using the “belief” notion in a wider way, it picks out something more akin to a natural kind. The notion becomes deeper and more unified, and is more useful in explanation (Clark and Chalmers, 1998, p. 14).

The next paragraph is devoted to a brief discussion of my interpretation of the role of coupling and of a minimal operational mark of the cognitive at play in the extended cognition framework.

### Some specifications on the coupling relation and a minimal operational mark of the cognitive

The main objection to the interpretation of the role of coupling that I have just proposed has to do with another standard criticism of the extended cognition framework: the risk of cognitive bloat, or the “overextension” of the cognitive (Rowlands, 2009, p. 2). Once we admit that causal coupling is sufficient for cognitive extension, are we not in danger of extending cognition too far, to phenomena that might not be cognitive at all? As a very quick reply to this objection, I propose two observations, concerning the qualification of the notion of coupling, and the search for the mark of the cognitive.

In the literature there have been at least two specifications of the “right” coupling, which leads to cognitive extension. The criteria proposed by Clark and Chalmers (see section The Extended Mind: A Problematic Boundary) restrict the relevant coupling relations to those characterized by reliability. Only those coupling relations that satisfy the three (or four, see fn. 2) criteria elaborated by Clark and Chalmers through a generalization of the Otto thought experiment—and later defined as the “glue and trust” criteria (Clark, 2010b)—can count as genuine clues to cases of cognitive extension. Basically, in addition to exhibiting a coupling relation, the connection between the agent and her alleged cognitive extension must be reliable and persistent over time.

The second specification on the coupling relation regards its actual function: it has been pointed out by Clark (2008) and later summarized by Shapiro (2011) in his accurate discussion of the case of gestures as actual examples of extended cognition (see Ch. 6). Gesture is characterized as a material structure that has a systematic cognitive effect on the listener as well as on the speaker (Goldin-Meadow, 2003). In this sense, the role of gesture seems similar to some forms of self-directed speech or writing for thinking (McNeill, 2005). The specific feature that distinguishes a mere causal impact from a coupled element that rightly enters the cognitive system is the fact that the latter is a self-generated output that the agent recycles as input in order to enhance the whole cognitive process. According to this view:

Gesture is *both* a systemic output and a self-generated input that plays an important role in an extended neural-bodily cognitive economy (Clark, 2008, p. 131).

In order to preserve both Otto's case and gestures as genuine examples of extended cognition, we can generalize this last condition stating that cases of extended cognition can be so described:

These are cases when we confront a recognizably cognitive process, running in some agent, that creates outputs (speech, gesture, expressive movements, written words) that, recycled as inputs, drive the cognitive process along. In such cases, any intuitive ban on counting inputs as parts of mechanisms seems wrong (Clark, 2008, p. 131).

It seems, then, that coupling alone is not sufficient to warrant cognitive extension: some further features need to be in play. The coupling relation must be reliable, and it must exhibit the just described condition of an output produced by the cognitive agent, recycled as input. With these two additional features in play, we seem to diminish the risk of cognitive bloat.

Of course, another way to escape the cognitive bloat objection would be to provide a solution to the mark of the cognitive. In fact, it seems that many problems raised by the extended cognition framework could be solved if we had an effective and non-committed mark of the cognitive to apply every time we wished to distinguish cognitive from non-cognitive processes. As we know, however, this objective is not so easy to achieve^13^.

In the next lines I would like to briefly present Clark's intuitions about the mark of the cognitive. We will see that in the extended cognition framework there is space for a minimal operational mark of the cognitive, one that is, of course, non-committed with reference to the localization of cognitive processes. In Clark's view, what is cognitive or non-cognitive is not the single component of a certain process, but the process as a whole, which must be involved in supporting intelligent behavior:

What makes a process cognitive […] is that it supports intelligent behavior (Clark, 2010b, p. 92).

Thus, according to Clark, the processes could in principle be implemented by various kinds of substances (biological or artificial neural substrates as well as external resources), because what defines something as cognitive is neither the substance that realizes it nor the detailed causal dynamics that characterize its workings. In this sense, cognitive processes in the extended cognition framework are individuated on the basis of coarse or common-sense functional considerations concerning cognitive processes such as memory, understanding, categorization, reasoning, etc.

It is the coarse or common-sense functional role that, on this model […], displays what is essential to the mental state in question (Clark, 2008, p. 89).

The reference to the causal relationship as the starting point of the analysis on mental reality is, after all, at the base of the (extended) functionalist intuition.

What makes some information count as a belief is the role it plays, and there is no reason why the relevant role can be played only from inside the body (Clark and Chalmers, 1998, p. 14).

The only available mark of the cognitive in the extended mind approach concerns the functional analysis of the resource in a given context. Thus, in the extended mind approach, the mark of the cognitive is not something already given; rather, it is something one construes and discovers, starting from an intuitive and shared idea of what a cognitive process is^14^.

## Conclusion: metaphysics, epistemology, and the mark of the cognitive

In this paper I have analyzed two of the main criticisms addressed to the functionalist version of the extended cognition thesis: the alleged lack of a mark of the cognitive, and the coupling-constitution fallacy. I have attempted to show that neither is as effective as it may appear at first glance.

The mark of the cognitive proposed by Adams and Aizawa does not rule out extended cognition, and confuses the typical with the normative.

The so-called coupling-constitution fallacy fails to recognize that the role of coupling in defining cognition, given a minimal mark of the cognitive, is exactly what extended cognition means to pursue in the first place, in accordance with its anti-individualistic spirit.

The approach to the study of the mind proposed in the extended framework thesis supports a dynamical ontology that moves from epistemological considerations toward the best possible explanation. Assigning to causal coupling a crucial role in “discovering” constitutive relations is a an approach that gives priority to epistemology rather than to metaphysics. In the extended approach, epistemology “guides” us, so to say, toward a revisionary metaphysics.

## Author contributions

The author confirms being the sole contributor of this work and approved it for publication.

### Conflict of interest statement

The author declares that the research was conducted in the absence of any commercial or financial relationships that could be construed as a potential conflict of interest.

## Footnotes

^1^Even if extended functionalism is more explicit on this point, it is true that, strictly speaking, functional individuation of mental states *per se* exceeds an organism's boundaries, unless one endorses a form of individualism (Sprevak, 2009). I will return to this point later (see fn. 6).

^2^The problematic nature of the fourth criterion was acknowledged early on by Clark and Chalmers themselves (Clark and Chalmers, 1998), and later by Rupert (2004, 2009). The criterion, however, has never been definitively defended or rejected, and its status remains suspended in the literature.

^3^Adams and Aizawa point out that these two hypotheses are to be kept distinct, because that of the system is less committing than the other, which refers to a common process (Adams and Aizawa, 2010a, p. 11). It is probably true that we can have systems composed of inhomogeneous parts, while “being part of the same process” might require more homogeneity among the participant entities.

^4^They refer to them as “hypotheses” or “clues,” but “conditions” or “criteria” would be more appropriate.

^5^Another alleged concession is discussed at length in Clark (2005, p. 6; Clark, 2010b, p. 86 ff.), and is what renders the requirement of non-derived representations in cognitive processes ineffective against extended cognition. See below.

^6^An important question tackled in the literature concerns the grain level at which functionalist explanations are articulated. However, some authors have claimed that functionalism *per se* implies extended cognition, in an even stronger form than that maintained by Clark and Chalmers (Sprevak, 2009; Wheeler, 2010). In order to block this derivation, one would have to endorse individualism, which restricts the relevant elements for cognition to the boundary of the individual. This would present a problem for Adams and Aizawa, who want to maintain functionalism, but do not want to deny extended cognition a priori.

^7^Adams and Aizawa (2010a, p. 91,92) acknowledge that the name “coupling-constitution fallacy” may not be the perfect name because it evokes the concept of constitution, which is not necessary to give rise to the fallacy. As an alternative, they suggest “coupling-extension fallacy,” which more directly addresses the problem of the relation between coupling and cognitive extension. In the end, however, they maintain the original name, and I will follow them in this.

^8^Adams and Aizawa (2009, 2010a) distinguish two versions of this fallacy to be found in the “simple coupling arguments”: based on simple coupling connections between an agent and his environment, and in the “system coupling arguments”—which require an intermediate step describing the causal relate as parts of a cognitive system. However, I do not think that this distinction is relevant in this context.

^9^For a detailed and thorough presentation and discussion about individualism in psychology, see Wilson (1995, 2004).

^10^This form of externalism is distinct from but clearly connected to semantic or content externalism, originally elaborated by Putnam through the Twin Earth thought experiment (Putnam, 1975). The difference is, so to say, the domain of application of the externalistic intuition: in the case of taxonomic externalism, the externalistic attitude is directed at the problem of how psychological science should individuate its objects of study, and not only to the problem of semantic meaning and reference.

^11^“Locational (i.e., vehicular) externalism is a stronger view than taxonomic externalism and entails it” (Wilson, 2004, p. 179).

^12^In Clark's words, even if not “organism bound”, human cognitive processing still remains “organism centered” (Clark, 2008, p. 123).

^13^For space reasons, I cannot engage in a thorough discussion of this important topic, which of course is a central question in the debate presented.

^14^Sure enough, our common-sense and intuitive idea of what is cognitive also depends on the work of contemporary science (cognitive psychology).
